# Progeny of old parents have increased social space in *Drosophila melanogaster*

**DOI:** 10.1038/s41598-018-21731-0

**Published:** 2018-02-27

**Authors:** Dova B. Brenman-Suttner, Shirley Q. Long, Vashine Kamesan, Jade N. de Belle, Ryley T. Yost, Rachelle L. Kanippayoor, Anne F. Simon

**Affiliations:** 1 Department of Biology, University of Western Ontario, London, ON Canada; 2 Department of Physiology and Pharmacology, University of Western Ontario, London, ON Canada

## Abstract

We report the effects of aging and parental age in *Drosophila melanogaster* on two types of responses to social cues: the choice of preferred social spacing in an undisturbed group and the response to the *Drosophila* stress odorant (dSO) emitted by stressed flies. The patterns of changes during aging were notably different for these two social responses. Flies were initially closer in space and then became further apart. However, the pattern of change in response to dSO followed a more typical decline in performance, similarly to changes in locomotion. Interestingly, the increased social space of old parents, as well as their reduced performance in avoiding dSO, was passed on to their progeny, such that young adults adopted the behavioural characteristic of their old parents. While the response to social cues was inherited, the changes in locomotion were not. We were able to scale the changes in the social space of parents and their progeny by accelerating or decelerating the physiological process of aging by increasing temperatures and exposure to oxidative stress, or via caloric restriction, respectively. Finally, when we aged only one parent, only the male progeny of old fathers and the progeny of very old mothers were more distant.

## Introduction

Social behaviours, defined as responses to another individual, are affected by several factors including previous experiences and genetic predisposition^1^. Much like other behavioural responses, social behaviours have been shown to change with age in organisms ranging from *Caenorhabditis elegans*^2^ and *Drosophila melanogaster*^3^, to humans^4^. For example, older honeybees (*Apis mellifera*) will forage outside the hive, whereas younger honeybees typically work inside the hive^5^ and older mice display decreased social contact in groups^6^.

Aging is the progressive deterioration of physiological function and fertility accompanied by an increased susceptibility to death^7^. There are many interconnected mechanisms of aging that lead to an aged phenotype and may influence conserved neural circuits^8,9^. One such mechanism is age-related variations in metabolism that can affect the individual through a gradual accumulation of metabolic by-products (such as reactive oxygen species or ROS) that can damage proteins^10^. Oxidative stress can also result in behavioural senescence because the brain is vulnerable to free radicals due to its high metabolic rate^8,9^.

Numerous behaviours have been shown to change in aging *Drosophila*. For example, in an analysis of various *Drosophila* behaviours from birth to death, walking, resting, feeding, and flying behaviours each declined with age and were correlated with time-of-death^11^. Similarly, both males and females displayed a change in negative geotaxis at 4 weeks of age. Other changes include increased activity at night and increased courtship behaviour at night in males^12,13^. Notably, not all behaviours change with age, for example the avoidance of an electric shock is stable throughout life^14,15^.

As well as its behaviour and physiology, the age at which an individual reproduces may affect its progeny. Studies focused mainly on mammals have shown, for example, reduced social interactions in mice with old parents^16^ and old grandfathers^17^. In humans, many of the studies on the effects of advanced parental age on social behaviour have taken place in the context of neuropsychiatric disorders^18^. For example, D’Onofrio *et al*. report that children conceived by fathers over the age of 45 are at a higher risk of developing disorders such as Autism Spectrum Disorders (ASDs) or Schizophrenia, which are characterised by altered social behaviour^19^. Of note, one example of the social deficit experienced by individuals with ASD is difficulty regulating personal space (or social spacing)^20^.

Although both males and females can contribute mutations to the next generation, the type of mutation introduced by each parent is often different. In humans, it has been suggested that issues arise from very young fathers due to fertilization with immature spermatids, whereas sperm of fathers who are over 45 years old have accumulated more *de novo* mutations^21^. Alternatively, older mothers contribute increased trinucleotide repeats to their progeny^21^. Interestingly, parental age affects the progeny in a sex-specific manner in *Drosophila melanogaster*^22^ where old mothers have shorter-lived daughters and, to a lesser extent, old fathers have shorter-lived sons^23^. However, the mechanism underlying this transgenerational effect has yet to be understood.

In this study, we used *Drosophila melanogaster* as a model to assess the changes with aging and possible transgenerational effects in simple social behaviours, including social spacing and the avoidance of the *Drosophila* stress odorant (dSO avoidance).

It has been well established that flies are social and respond to others in a group in a non-random manner (see Ramdya *et al*.^24^ for a recent review). Although studying social group formation and group behaviour began as early as 1961 for *Drosophila*^25^ and specifically in 1975 for the species *melanogaster*^26^, this field has recently gained momentum^24^. One of the decision-making processes that takes place in individuals within a group, including flies, is the establishment of a preferred distance among neighbours, or social spacing, which is generated by a balance between attractive and repulsive social cues^27–30^. Studies of social spacing in *Drosophila melanogaster* in the past five years have led to the observations that social spacing is indeed not random^27,28^, and changes in response to social experience (such as mating and isolation^8^). In terms of modalities, vision is necessary for social spacing, but classical olfaction is not^27,28^. From these findings and others, a neural circuit involving dopaminergic signalling^31^ and cholinergic neurons of the mushroom body^27^ (possibly downstream of those dopamine neurons) is emerging as a modulator of social spacing. At the synaptic level, Neurobeachin (an anchor protein^32^) and Neuroligin (a cell adhesion protein^33^) are also implicated in social space. Both of those postsynaptic proteins have human homologues that are associated with autism. Finally, Bisphenol A, a toxin that is known to disrupt neurodevelopment (including probably in *Drosophila*^34^) and is linked to autism, also leads to abnormal social spacing^35^.

In another type of response to social cues, flies strongly avoid the volatile substance dSO emitted by stressed flies^36,37^. CO_2_ has been identified as one of the compounds in dSO^37^, although other unidentified compounds are required to elicit the full avoidance of dSO^37,38^. Because dSO is emitted by stressed flies and induces a response from conspecifics, it is considered a social cue. Compared to social space, dSO avoidance requires different sensory modalities such as olfaction. Further, dopaminergic neuromodulation is not involved^31^.

Although some complex social behaviours are known to change with aging, such as courtship^39^ and aggression^40,41^, the effect of age on very different and quite simple social behaviours, such as the preferred social spacing between individuals and the response to dSO, has yet to be investigated. In this study, we characterise these changes and report that, contrary to effects on locomotion, the effect of age on social space and dSO avoidance are inherited by the next generation. The effect of individual and parental age on social space can be accelerated with increased temperature or paraquat exposure and is prevented by caloric restriction. We also observe increased social space with age in another laboratory strain, suggesting this phenomenon is generalizable.

## Results

### Unlike locomotion and dSO avoidance, the changes with aging observed in social space are dynamic

#### How old is old?

In order to perform studies on aging, we first generated survival curves of Canton-S, our laboratory strain, to determine when flies start to die (Supplemental Fig. 1A). Under our laboratory conditions, 100% of flies were alive at 7 days and we used this age as our “young” control, as they are sexually mature, and do not display declines in behaviour^14^. The oldest flies we tested were 50 days old (when 50% of the cohort were still alive), but it was difficult to obtain progeny from those old flies (see fertility curve Supplemental Fig. 1A). Thus, for most of the study, 30 day-old individuals represented old flies, as they had reduced survival (90% of the cohort was alive), while remaining fertile (Supplemental Fig. 1A).

#### Social space

**Figure 1:**
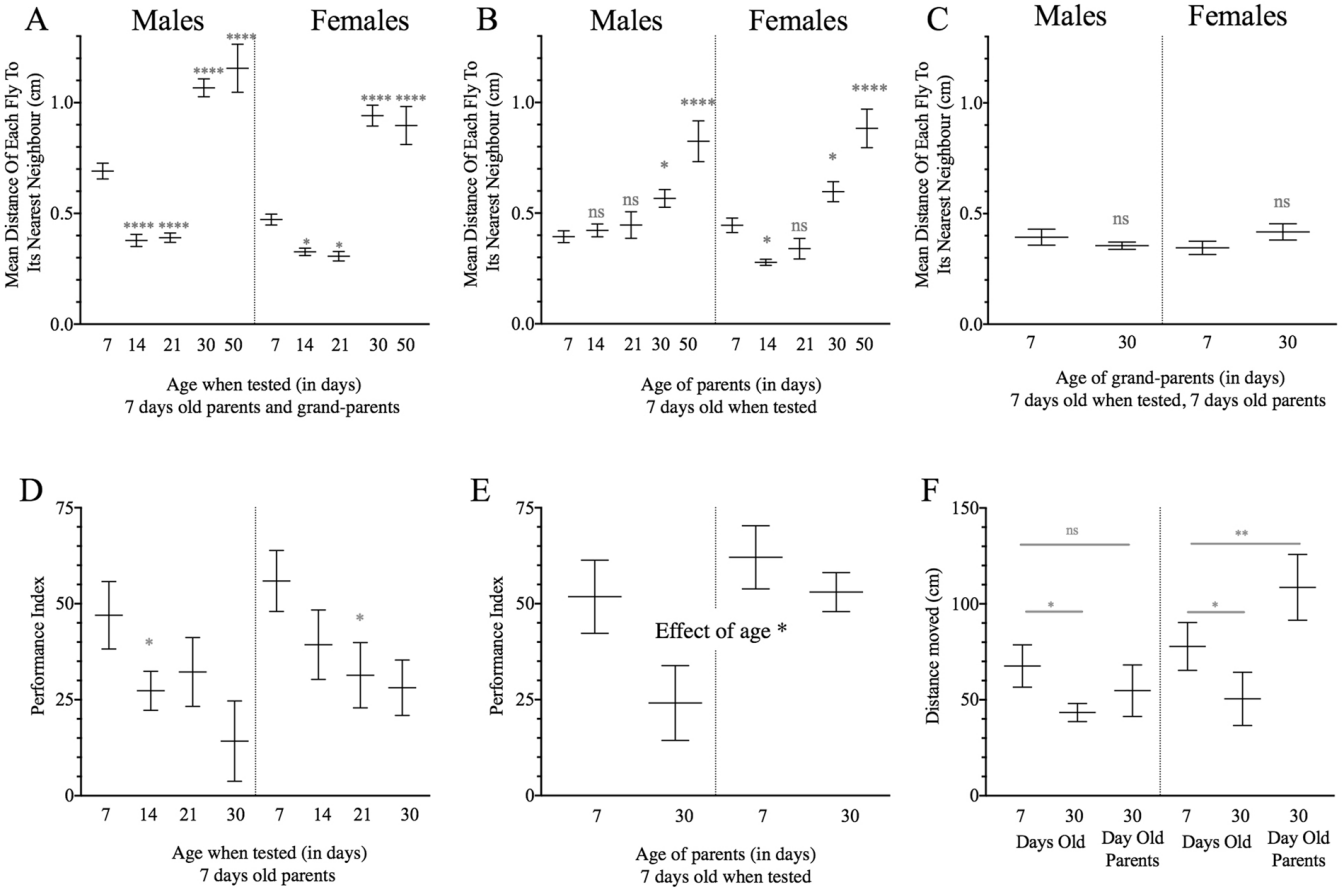
Social space, dSO avoidance and locomotion of aged *D. melanogaster* and their young progeny. (**A**–**C**) The mean distance of each fly to its nearest neighbour (mean ± s.e.m.) in the social space assay (n = 9; one-way ANOVA and Holm-Sidak *post hoc* test or unpaired, one-tailed student t-test; for two-way ANOVA: see Results). (**A**) Effect of aging: 14 and 21 day-old flies are closer to their nearest neighbour, whereas 30 and 50 day-old flies are further, as compared to 7 day-old flies in both sexes. (**B**) Effect of parental age: The young (7 day-old) male progeny (G1) of 14 and 21 day-old parents are as close as the young progeny of 7 day-old flies, whereas young female progeny of 14 day-old flies are closer to their nearest neighbour. However, both the young male and young female progeny of 30 and 50 day-old flies are further apart. (**C**) No effect of the grandparental age: The 7 day-old second generation (G2) of 7 and 30 day-old parents do not differ in the distance to their nearest neighbour. (**D**,**E**) Performance index (PI) of dSO avoidance (n = 9; one-way ANOVA and Holm-Sidak *post hoc* test). (**D**) Effect of aging: flies older than 7 days have a lower PI for both male and females and are not avoiding dSO as efficiently as young flies (see text for two-way ANOVA). (**E**) Effect of parental aging: The 7 day-old progeny of 30 day-old flies have a lower PI than the young progeny of young parents (n = 8; two-way ANOVA and Holm-Sidak *post hoc* test, F_1,32_ = 4.84, p = 0.0352). (**F**) Locomotion 30 day-old males and females move shorter distances than 7 day-old individuals (effect of age in a two-way ANOVA and Holm-Sidak *post hoc* test; F_1,27_ = 5.48, p < 0.027, no significant differences among the sexes and no interaction between aging and sex). However, 7 day-old male progeny of 30 day-old parents were not different from one another, but the 7 day-old female progeny move longer distances (interaction between sex and age of parents: F_1,25_ = 8.38, p < 0.0078). n in legend = number of replicates, and for all graphs: ns = not significant, *p < 0.05, **p < 0.01, ***p < 0.001, and ****p < 0.0001.

To fully characterise the effect of aging on social spacing, using 7 day-old parents, we first assessed the effect of various ages of their progeny (7, 14, 21, 30 and 50 days old; Fig. 1A). The mean distances to the closest neighbor varied with both age and sex (F_4,122_ = 89.29, p < 0.0001 and F_1,122_ = 21.99, p < 0.001, respectively, in a two-factor analysis). But the pattern of changes with age was dynamic. Specifically, 14- and 21-day-old flies were closer to their nearest neighbour as compared to the 7-day-old young control flies. However, by 30 and 50 days old, both males and females were further from their nearest neighbour as compared to 7 day-old flies (one-factor analysis in males: F_4,61_ = 43.95; p < 0.0001, and females: F_4,61_ = 47.32; p < 0.05). Therefore, in both males and females, social distance is minimal between the ages of 14 and 21 days and maximal between the ages of 30 and 50 days.

#### dSO avoidance

As expected, since dSO avoidance is a type of response to olfactory social cues, the changes in performance with age were more typical of olfactory cues^15^. Indeed, dSO avoidance declined with age (F_3,73_ = 4.86, p = 0.0039; Fig. 1D), and there was no difference between sexes (F_1,73_ = 2.40, p = 0.1255).

#### Locomotion

Similarly to what we and others have reported^14,15^, locomotion declined with aging (Fig. 1F). Overall, increased age leads to a decrease in the distance travelled (F_1,27_ = 5.48, p < 0.027) in both sexes.

### The increased social space of the old age phenotype is passed on to the youth of the next generation

We compared the 7 day-old progeny of young and old parents to test if there was potential transgenerational influence on these behaviours.

#### Social space

Parental age affected social space of the next generation (F_4,71_ = 32.90, p < 0.0001; Fig. 1B). Specifically, 7 day-old male and female progeny of 30 and 50 day-old flies were further apart (F_4,31_ = 12.53, p < 0.05 and F_4,37_ = 22.47, p < 0.0001, respectively). Additionally, the female progeny of 14 day-old parents were closer to their neighbours (p < 0.05). This transgenerational effect was not observed after the first generation, as we found no differences in social spacing in the 7 day-old progeny from 30 day-old grandparents (Fig. 1C), in both males (t = 1.053, df = 14, p = 0.1551) and females (t = 1.518, df = 14, p = 0.0757). We tested up to the fifth generation with similar results (Supplemental Fig. 2A–C).

#### dSO avoidance

Increased parental age also led to a reduced dSO avoidance in their young progeny similar to that seen in old flies (effect of age F_1,32_ = 4.84, p = 0.0352 - Fig. 1E).

#### Locomotion

Because we found an inheritance of the behavioural response of aged parents in their progeny with group behaviour, we wanted to see if there was a similar response in locomotion, which is a non-social, individual behaviour. We found that an older age of parents (30 days) affected the distance that their progeny moved, but only in females, who moved longer distances in the same time frame (interaction between sex and age of parents: F_1,25_ = 8.38, p < 0.0078; Fig. 1F).

#### Life-history traits

To assess the extent to which these transgenerational effects in the progeny could be due to an overall altered physiology, we investigated non-behavioural traits such as fertility (number of eggs laid per female), fecundity (number of adult progeny developed from those eggs) and survival (males and females). Neither fecundity nor fertility of females with 30 day-old parents was reduced but longevity was increased. As was found for social space, this change in longevity returned to the control baseline in the second generation (Supplemental Fig. 1 and Supplemental material for data and statistical analysis).

Since the transgenerational effect was more pronounced in measures of social space than for dSO avoidance, we focused the remaining studies on social space.

### One old parent is sufficient to pass on the increased social space of old age to the young of the next generation

To see if the transgenerational effect was due to the influence of both parents, we next tested the effect of one older parent on the social space of its progeny.

#### Male progeny of 30-day-old fathers have increased social space

**Figure 2:**
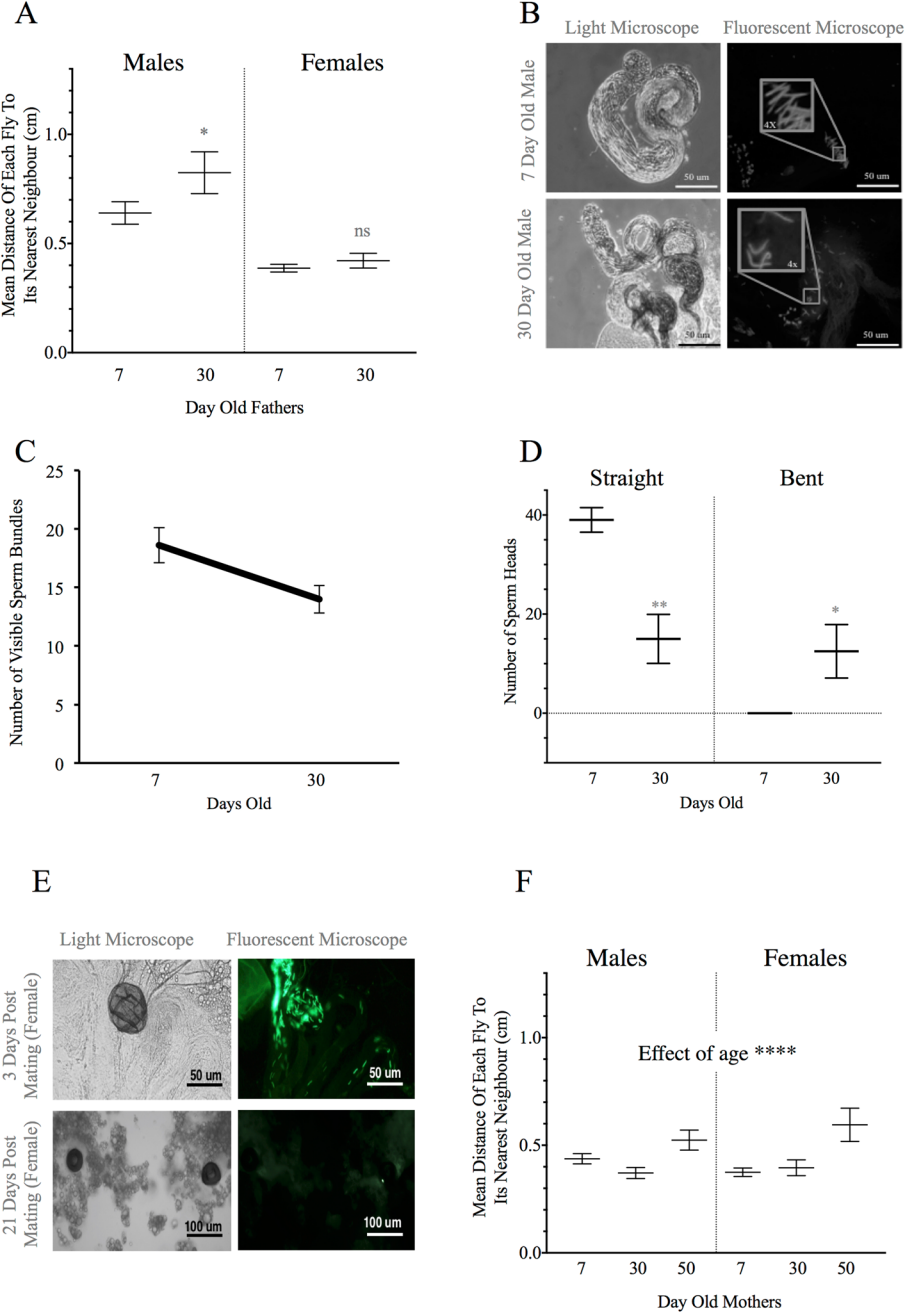
Social space and sperm morphology when only one parent is aged. (**A**–**D**) old father. (**A**) The mean distance of each fly to its nearest neighbour (mean ± s.e.m.) in the social space assay (n = 9; unpaired, one-tailed student t-test, for two-way ANOVA see Results). The 7 day-old male, but not female progeny, of 30 day-old fathers are further from their nearest neighbour. (**B**) Testes of 30 day-old (lower panel) males appear thinner and darker under light microscopy, with (**C**) fewer sperm bundles (14 ± 1.18) as compared to 7 day-old males (upper panel; 18.6 ± 1.50; mean ± s.e.m.; n = 10). (**D**) There are fewer individual straight sperm heads in 30 day-old males (15 ± 4.95) as compared to 7 day-old males (39 ± 2.48 straight; p < 0.01) and there are no bent sperm heads at 7 days but there are 12.5 ± 5.39 bent sperm heads at 30 days of age (n = 10; two-way ANOVA and Holm-Sidak *post*-*hoc* test). (**E**,**F**): old mothers. (**E**) GFP-tagged sperm is no longer visible in the spermatheca and reproductive tract of 30 day-old females. Upper panels show the presence of GFP-tagged sperm in females 3 days post-mating with males containing GFP-tagged protamine sperm and lower panels show the absence of this fluorescent sperm 21 days post-mating (n = 5). (**F**) The mean distance of each fly to its nearest neighbour (mean ± s.e.m.) in the social space assay (n = 9, two-way ANOVA main effect of age: F_2,64_ = 12.46, p < 0.0001). The young progeny (G1) of 30 day-old mothers display the same social space as the young progeny of 7 day-old mothers. However, both female and male progeny of 50 day-old mothers were significantly further from their nearest neighbour. n in legend = number of replicates, and for all graphs: ns = not significant, *p < 0.05, **p < 0.01, ***p < 0.001, and ****p < 0.0001.

When old males (30 day-old) were mated with virgin females, their 7 day-old male, but not female, progeny had an increased social space (effect of sex: F_1,32_ = 35.78, p < 0.0001; Fig. 2A).

#### Older males show differences in number of sperm bundles and sperm morphology

To begin to understand how the age of the fathers might affect their progeny, we focussed on the sperm, as this is the material inherited from the male. Under both bright-field microscopy and fluorescent microscopy we observed differences in the number of sperm bundles, testes and sperm head morphology between young and old males (Fig. 2B). Testes of old males were thinner and darker (Fig. 2B, bottom, left panel), perhaps due to reduced sperm content within the testes. Indeed, 7 day-old males had more sperm bundles per testis (18.6 ± 1.5), compared to 30 day-old males (14 ± 1.2; mean ± s.e.m.; n = 10; Fig. 2C). We used DAPI (a fluorescent dye that binds to AT-rich regions) to visualize the contents released by the testes (Fig. 2D). The total number of sperm heads was not statistically different among age groups (mean ± s.e.m. for 7, 30 and 50 day-old, respectively: 39 ± 2.5, 28 ± 10 and 28 ± 11). However, we counted more individual straight sperm heads in 7 day-old males and more bent sperm heads in both 30 day-old and 50 day-old males (sperm head morphology F_1,12_ = 28.82; p = 0.0002; and interaction of sperm head morphology and age F_1,12_ = 22.30; p = 0.0005). Therefore, our data suggest that the testes and their contents deteriorate with age.

#### Young progeny from 50, but not 30, day-old mothers have increased social space

Virgin females were mated with young males that had sperm containing GFP-tagged protamines that could be visualized under fluorescent microscopy. After three days of mating, the sperm of young males were seen in the female reproductive tract (Fig. 2E, top panels). After three weeks isolated from males, the sperm were no longer visible in the female reproductive tract (Fig. 2E, bottom panels). We were thus able to confidently obtain the progeny of old female flies and 7 day-old male flies, without any interference of remaining sperm from other males.

Maternal aging to 50 days led to increased social space in both sexes (F_2,64_ = 12.46, p < 0.0001; Fig. 2F). This differs from the paternal effect, which was significant only in the male progeny of 30 day-old fathers. However, the effect of maternal age was more pronounced for the female progeny, as detected in a one-factor analysis, which led to significance in the female progeny of 50 day-old mothers (F_2,28_ = 8.402; p < 0.0001), but not in the male progeny (F_2,36_ = 5.493; p = 0.1185).

### Manipulation of the aging process recapitulates the age and transgenerational effects

We next aimed to confirm that the atypical pattern of change in social space with aging was indeed due to senescence. We therefore accelerated or decelerated the aging process and tested whether the pattern would change accordingly, and whether those changes could be passed on to the progeny.

#### Accelerated aging through increased temperature

**Figure 3:**
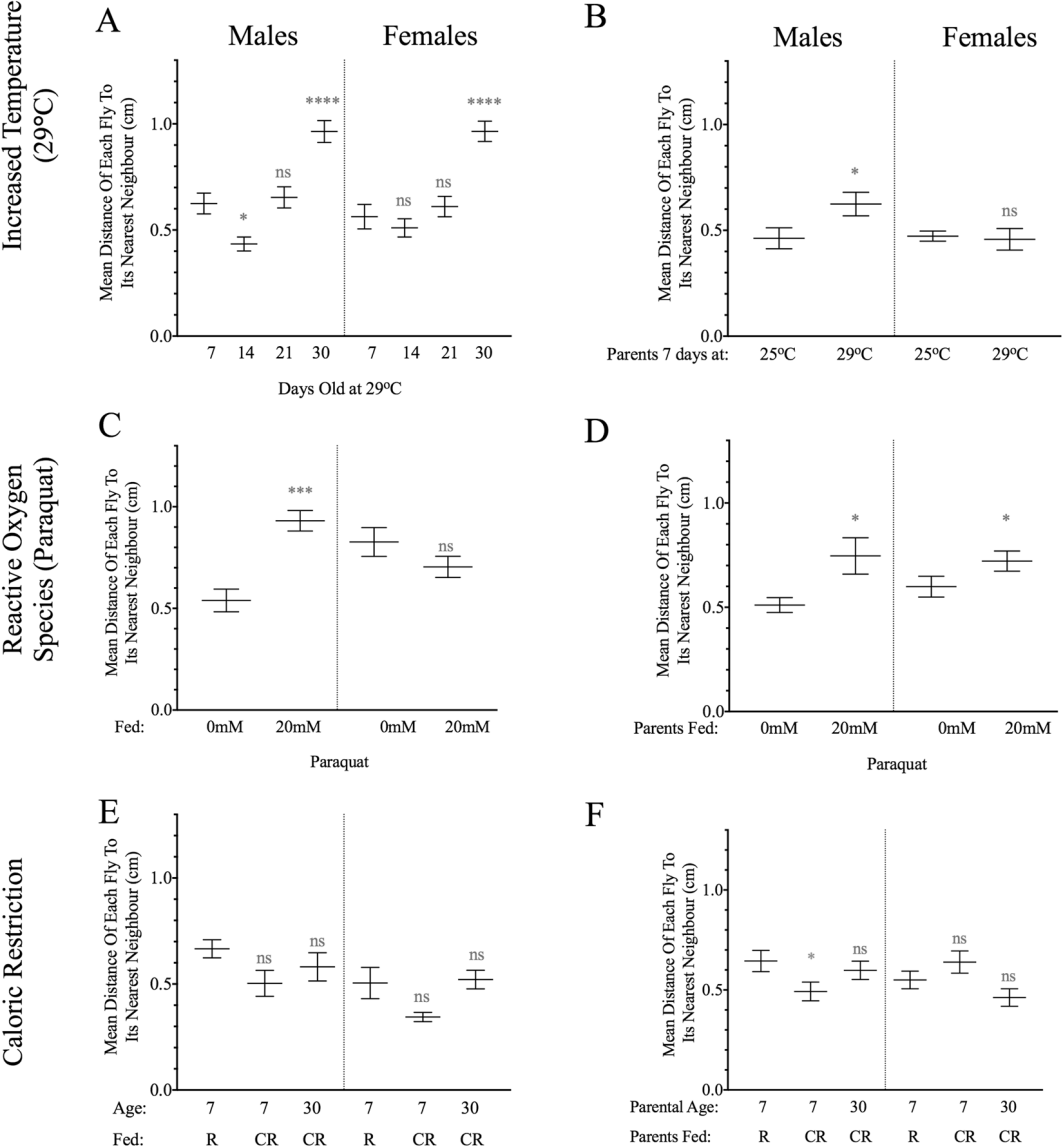
Effect of environmental manipulations to accelerate or decelerate aging on social space. Data are shown as mean distance of each fly to its nearest neighbour (mean ± s.e.m.). (**A**,**B**) Effect of temperature (n = 9; one-way ANOVA and Holm-Sidak *post hoc* test, for two-way ANOVA see text). (**A**) Effect of being aged at 29 °C: As observed at 25 °C, 14 day-old male flies that are exposed to 29 °C are closer to their nearest neighbour, and both males and females are further from their nearest neighbour at 30 days-old. But there was no difference between 7 days and 21 days old flies, contrary to what was observed at 25 °C. (**B**) Effect of parents aged at 29 °C: Flies were raised to 7 days at 25 °C but their parents were aged to 7 days at either 25 °C or 29 °C. Only 7 day-old male progeny of parents at 29 °C are further from their nearest neighbour. (**C**,**D**) Effect of paraquat (n = 9; unpaired, one-tailed student t-test, for two-way ANOVA see text). (**C**) Effect of feeding paraquat: Only male flies that were fed 20 mM paraquat were further from their nearest neighbour than individuals who were not fed paraquat (all flies tested at 7 days old + 13.5 hours post-feeding either 0 mM or 20 mM). (**D**) Effect of parental feeding: The unexposed 7 day-old progeny of *D. melanogaster* fed 20 mM paraquat were further to their closest neighbour, although only the male progeny were significantly further apart. (**E**,**F**) Effect of caloric restriction (CR ; n = 9; one-way ANOVA and Holm-Sidak *post hoc* test, for two-way ANOVA see text). (**E**) Effect of age on CR: Flies aged to 7 or 30 days on caloric restriction food are as close to their nearest neighbour as flies fed regular food for 7 days (**F**) Effect of parental age on CR: The 7 day-old (young) male progeny of young flies fed CR food are closer to their closest neighbour than the progeny of 30 day-old flies fed CR. In females, the young progeny of young flies fed CR appeared slightly further apart whereas the progeny of 30 day-old flies fed CR appear slightly closer to their nearest neighbour, although this distance was not significant. n in legend = number of replicates, and for all graphs: ns = not significant, *p < 0.05, **p < 0.01, ***p < 0.001, and ****p < 0.0001.

As *D. melanogaster* does not internally regulate its body temperature^10^, one way to manipulate its metabolism is to adjust the environmental temperature. Higher temperatures can speed up metabolism^10^. Because survival decreases quickly at 29 °C (Supplemental Fig. 3B), we were unable to test individuals at 50 days old, but we tested the social space of flies that were aged at 29 °C for 7, 14, 21, or 30 days. As seen at 25 °C, 14 day-old males were closer to their nearest neighbour (F_3,36_ = 20.62; p < 0.05) compared to 7 day-old controls, whereas 30 day-old males and females aged at 29 °C were further from their nearest neighbour (F_3,36_ = 20.62, p < 0.01 and F_3,34_ = 17.13, p < 0.0001, respectively; Fig. 3A). However, contrary to what was observed at 25 °C, the 21 day-old flies were further apart than the 14 day-old flies, as would be expected with an acceleration of the aging process.

Also, the effects of increased temperature on parental aging were passed on to the next generation. When the progeny of 7 day-old parents aged at 29 °C were allowed to develop and age for 7 days at 25 °C, male progeny (t = 2.11, p = 0.0253, df = 16), but not female progeny (t = 0.27, p = 0.397, df = 14), were further from their nearest neighbour as compared to the 7 day-old progeny of parents aged at 25 °C (Fig. 3B).

#### Accelerated aging through increased ROS production

Another way to accelerate the biological aging of *D. melanogaster* is to feed flies the ROS generator, methyl viologen (paraquat^42^ - see methods and Supplemental Fig. 3C for survival curves and dose effect of exposure to paraquat). Only male, but not female, flies that were fed 20 mM of paraquat were further from their nearest neighbour (t = 5.15, df = 15, p < 0.0001; Fig. 3C). Therefore, only males had an accelerated aging pattern. This is not surprising as females have been shown to be more resistant to stressors, such as desiccation and starvation^43^. As females are bigger, and as males and females invest different amounts of energy into gamete production, the build-up of ROS may be imbalanced and thus an individual’s threshold to withstand such stress may also differ^44^. In contrast, a transgenerational effect was seen in both male and female progeny of parents that were exposed to 20 mM paraquat and were further from their nearest neighbour as compared to the progeny of parents that were not fed paraquat (main effect of paraquat: F_1,27_ = 8.913; p = 0.006, and t-test in male t = 2.37, df = 13, p < 0.0001 and in female t = 1.76, df = 14, p = 0.0500 - Fig. 3D). Therefore, this result alludes to different mechanisms by which ROS affects both the soma and gametes.

#### Decelerated aging through caloric restriction

A caloric restriction (CR) diet limits the amount of harmful metabolic by-products of aging^45^. Consistent with our aging results, we observed no differences in the social spacing between individuals aged to 7 days on regular food or 30 days on CR in both males and females (Fig. 3E). We then allowed flies that were aged to 7 or 30 days on caloric restriction to lay eggs for 2–3 days on regular food. As expected, there was no increase in social space between the progeny of individuals aged to 7 days on regular food or 30 days on CR, and the females were actually closer to their nearest neighbour (F_1,19_ = 6.12; p = 0.0229; Fig. 3F). Overall, the social space assay revealed that the young progeny of old parents on CR were no more distant, much like their parents.

Therefore the CR diet was able to mitigate the age-related effects of damaging metabolic by-products, which then prevented changes to social spacing behaviour. In rats, similar alterations to parental diets have been shown to affect the behaviour of their offspring^46^. Of note, we see an effect on behaviour without an impact on longevity (Supplemental Fig. 3C), which may indicate the CR diet was a moderate caloric restriction^10^.

### Increased social space with age in another lab strain: Oregon-R

**Figure 4:**
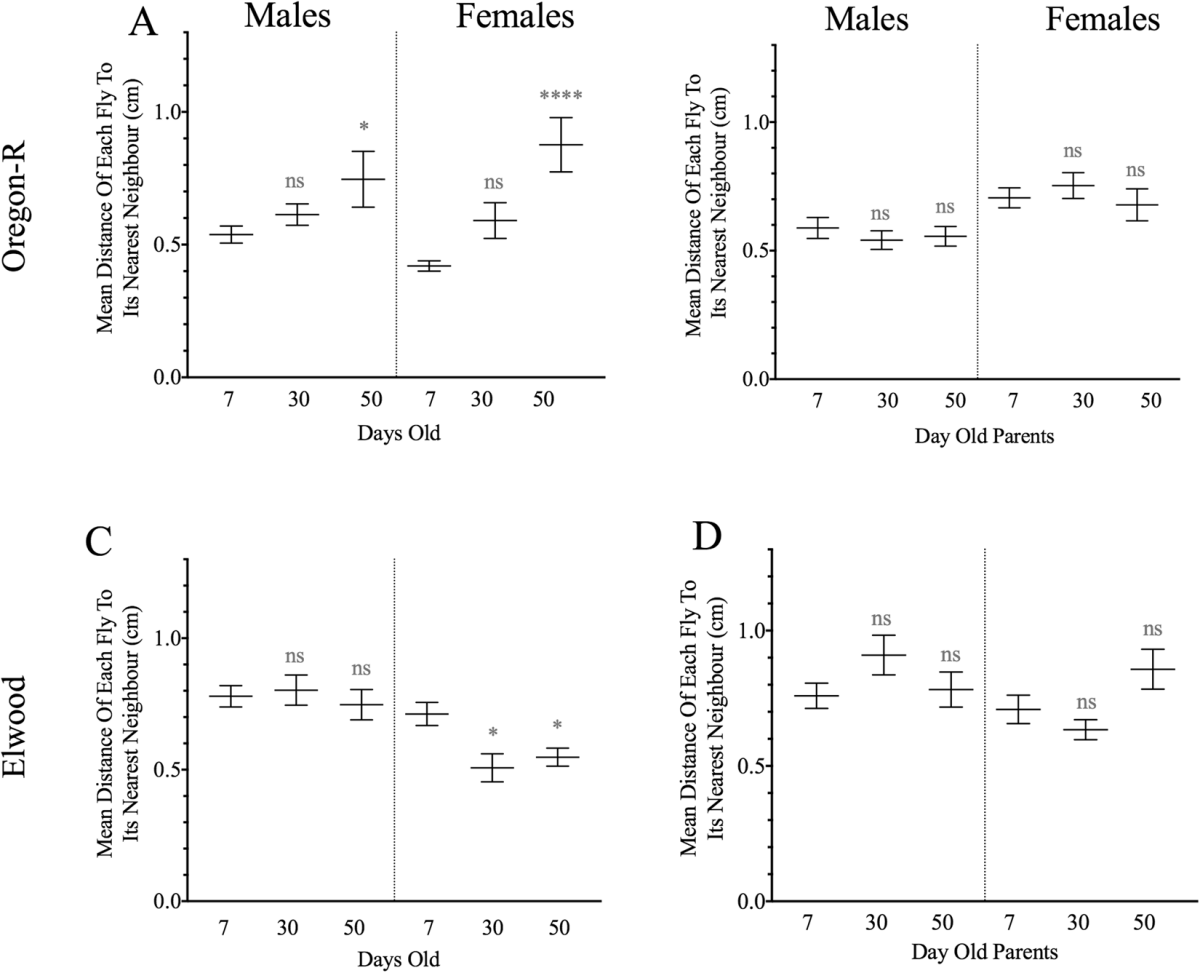
Transgenerational effect of old age on social space in different genetic backgrounds: Changes to social space with aging is found in Oregon-R. Data are shown as mean distance of each fly to its nearest neighbour (mean ± s.e.m.) in the social space assay of different strains (n = 9; one-way ANOVA and Holm-Sidak *post hoc* test, for two-way ANOVA see text). (**A**) Effect of aging in Oregon-R. Males and female are further apart at 30 and 50 days old. (**B**) Effect of parental age in Oregon-R. The male and female progeny of 30 or 50 day old Oregon-R parents are not more or less close. (**C**) Effect of aging in Elwood. Males are not further apart at 30 or 50 days of age. Female at 30 and 50 days old are closer than those at 7 days, unlike other strains. (**D**) Effect of parental age in Elwood. As for Oregon-R, the male and female progeny of 30 or 50 day old Elwood parents are not more or less social. n in legend = number of replicates, and for all graphs: ns = not significant, *p < 0.05, **p < 0.01, ***p < 0.001, and ****p < 0.0001.

We wanted to assess whether there was a genetic underpinning to the effect of age on social space. We therefore tested two additional genetic backgrounds including another lab strain, Oregon-R, and a recently caught wild-type strain called Elwood. Similar to Canton-S flies, Oregon-R flies were further apart at older ages, in both males (F_2,28_ = 3.83, p = 0.0338) and females (F_2,33_ = 16.9, p < 0.0001; Fig. 4A). The progeny of aged parents, however, did not follow the same pattern (Fig. 4B). The recently caught strain Elwood showed a different social spacing pattern; Elwood females were closer to their nearest neighbour with increasing age, while aging did not affect Elwood males (F_2,31_ = 5.60; p < 0.05; Fig. 4C). Additionally, the male and female progeny of old parents tended to be further apart, although the difference was not statistically significant (Fig. 4D). Therefore, we found that there are genetic aspects to both the effect of individual age and age of the parents on social space, which might have been selected for in laboratory strains.

## Discussion

*Drosophila melanogaster* Canton-S displays dynamic changes in social spacing with age, where individuals move closer to one another for the first two to three weeks of age, then move further apart. The effect of old age on social spacing is transmitted to the young of the first generation. We were able to accelerate or decelerate both the effect of aging and the transgenerational inheritance on social space through manipulation of the physiological aging process. Old fathers induced a change in social space in their sons but not their daughters and very old mothers induced a change in behaviour in both male and female progeny. The reduced avoidance of dSO, another social cue, was also transmitted to the progeny. This age-related decline in performance in dSO avoidance is similar to what has been shown with a change in response to olfactory alterations or motor function with aging^47^. Importantly, the progeny of old parents were not negatively impacted overall because their performance in a non-social behavioural assay (locomotion) and their survival did not decrease. The lack of correlation among the changes in locomotion, dSO avoidance and social space indicates that the changes to dSO avoidance and social spacing are not due to locomotor differences, consistent with what was reported previously^28,29,35^.

These age-related changes in social space could be due to several factors. When flies were two and three weeks old, both males and females were slightly closer. Interestingly, females at this age were also the most fertile (supplemental Fig. 1A). Pheromones such as cVA, which is produced by males and acts as a repellent to other males^48^, and is transferred to females during copulation, could be affecting social spacing during this highly fertile period^49^, resulting in closer social space. Similarly, when individuals are highly fertile, they may be more social as they are looking for sexual partners. However, we found no correlation between changes in fecundity or fertility and behaviour (r = 0.1725 each, p = 0.126).

At older ages, both males and females were further apart. In this assay, individuals are interacting only with the same sex, who may not be providing them with any specific benefit, and thus there is no incentive to being closer together. Alternatively, they may become indifferent to social interactions at older ages. Finally, declines in motor function or sensory modalities may be causing a change in the social behaviour of aged flies.

The effect of old age on increasing social space was transmitted to the next generation, but did not extend past the first generation. Because there is no overlap of the generations, and the fertilized eggs are deposited onto the food, alteration to the gametes during the aging process is the only possible explanation for our observations. Additionally, those gametes could be altered in different ways.

First, accumulation of random mutations occurring throughout aging would likely fall on longer genes^50^. Interestingly, many autism-associated genes are very long, and tend to be synaptic genes^51^. Such genes are likely to affect social behaviour such as social spacing and dSO avoidance. The transgenerational effect of aging of the decreased dSO avoidance indeed supports the idea that the decline in performance with parental age could be due to mutation accumulation within the parental gametes. However, this cannot explain the pattern observed for social space. This transgenerational inheritance also cannot be a learned behaviour from the parents, as the different generations were never co-habiting and thus social entrainment of behaviour, as seen previously in *Drosophila*^52,53^, could not have occurred.

Beyond the genome, age-related modification in the gametes could affect macromolecules such as certain types of RNA^54^ or histones. Further studies will be necessary to precisely identify the mechanisms of this transgenerational effect. Although the impact of age appears much stronger when both parents are old, the sex-specific bias in the inheritance can also inform us as to what known age-related mechanisms may be affecting and altering the gametes. Previous studies have shown a sex difference in the behaviour of progeny of aged mothers or fathers, and we saw a similar phenomenon^22^. Fathers contribute very little cytoplasm and thus genomic material is likely what is damaged and causing changes in the progeny. Additionally in *D. melanogaster*, there is little to no methylation of the genome and thus epigenetics via methylation is likely not causing this change^55^. We do show a compelling piece of evidence suggesting that male genetic and other epigenetic material could be damaged. When visualised by fluorescent microscopy, the sperm heads are bent in older males. This abnormal shape is probably due to the improper exchange of histones for protamines when the genome is compacted. In fact, removal of the genes for protamines lead to 20% of sperm heads having a bent shape in *D. melanogaster*, while still remaining fertile^56^. Improper compaction of the genome can leave areas of the DNA exposed to stressors such as ROS, thus leading to damage that will affect the next generation. Additionally, incomplete exchange of histones for protamines, as seen in both humans and mice, will result in the transmission of post-translational modifications on histones, an epigenetic mechanism, which can also affect the progeny^57^. Interestingly, the female progeny of aged fathers were somehow resistant to the damage provided by older males. However, more work is needed to determine which factor, namely sex chromosomes or sex hormones, has a larger impact on the behaviour of females with old fathers.

While aging fathers for 30 days was sufficient to affect their offspring, this was not the case for the mothers, who must be aged further to observe changes in the progeny, suggesting a different sensitivity to the aging process in female gametogenesis. The female fly genome is not compacted in gametes and thus histones are passed on to the progeny along with epigenetic modifications^58^. The large egg also contains other transmissible elements such as non-coding RNA and microRNAs, both of which can change with aging and have each been shown to affect progeny development^59^. Again, here, more work will need to be performed to determine which factors, specifically genetic or epigenetic factors, are responsible for the alteration of behaviour in the progeny of old females.

Finally, Oregon-R, another lab strain of *D. melanogaster*, also exhibits an increase in social space with aging. However, a recently caught strain, Elwood, did not show any changes. Perhaps years of captivity within the lab resulted in the selection of this age-dependant trait. In any case, this strain-specific effect suggests a genetic influence of the phenomenon that we report.

Taken together, our results demonstrate transgenerational effects on social behaviours in an animal model with powerful genetic tools, which will allow us to elucidate the underlying mechanisms that may be evolutionarily conserved, including mechanisms that result in human disease. Indeed, recent studies have linked neuropsychiatric disorders such as Autism Spectrum Disorders (ASDs) and Schizophrenia to old fathers. These disorders often include changes in social behaviours such as inappropriate social distance, and we demonstrate that mechanisms regulating social space and its transgenerational inheritance can be effectively modeled in *Drosophila melanogaster*.

## Methods

### Fly stocks and rearing conditions

#### Fly stocks

*Drosophila melanogaster* Canton-S, Oregon-R and Elwood strains are from our own stocks. Apart from the experiments with caloric restriction food, the strains were reared in mixed sex in bottles over Jazz-Mix^TM^ media (brown sugar, corn meal, yeast, agar, benzoic acid, methyl paraben and propionic acid; Fisher Scientific; 25 °C, 50% humidity with a 12:12 light: dark cycle, lights on at 8 am). Bottles of flies with fresh food were made bi-weekly when the parents were less than 7 days old, with approximately 200–300 mixed-sex flies per bottle (6 oz bottle with 50 mL of food) or 40 flies per vial (2.85 × 9.5 cm containing roughly ~2 cm high of food). Flies were removed regularly to prevent new emerging flies cohabitating with their parents.

#### Caloric restriction

Upon collection from stock bottles (above), flies were placed in vials containing food with low yeast/low sucrose content, as this was found to be the most efficient combination of adjusting sugar and protein (yeast) to reduce the calories by 30–40% in *D. melanogaster* (recipe adapted^60^, 50.8 kcal/ 100 ml medium in CR, 146.8 kcal/100 ml in Regular (R) Jazz-Mix^TM^ media). Every 2–3 days, flies were transferred to CR at 25 °C.

### Generating old flies and the progeny of old flies

#### Generating old flies

Once a week, 1 to 3 day-old flies were collected from bottles (40 flies/vial, 7 vials/week) under cold anaesthesia. Flies were then aged to constitute an aging collection. Aging flies were transferred to new media in vials every 2–3 days.

#### Generating the progeny of old flies

When the aging flies reached 90% survival (30 ± 1.53 days; Supplemental Fig. 1A), their progeny were saved and allowed to develop to adulthood (first generation). At one week old, the progeny of this first generation were collected and allowed to develop to adulthood and were also used for behavioural tests (second generation). This cycle of maintaining flies and collecting the progeny of both young and aged flies continued for several generations (second, third, fourth and fifth generations of 7-day-old “young” flies and 30-day-old “old” flies) and were tested with the social space assay.

#### Generating the progeny of old fathers

30 day-old males were separated from females under cold anaesthesia and were set aside for mating with virgin females (5 males and 5 females per vial; 3 vials were collected per week for 3 weeks for a total of n = 9 vials or biological replicates). 3 days later, all flies were removed from the vials and the resulting eggs were allowed to develop to adulthood and were used for behavioural testing compared to the progeny of 7 day-old male flies mated with virgin females.

#### Generating the progeny of old mothers

Virgin female Canton-S *D. melanogaster* were collected under cold anaesthesia and mated for 3 days to males with GFP-tagged sperm^61^ (n = 20 virgin females, 5 GFP-tagged males, n = 8 vials per week). Females were mated prior to aging because older virgin females have been reported to have lower sexual receptivity; the initial mating should prevent this when they are eventually mated with the new males. We used males with GFP-tagged sperm so that we could track, using fluorescent microscopy, how long females retain sperm from the initial mating event. When females did not have any visible sperm, we mated them with 7 day-old males for several days before removing the males from the vial (10–20 females, 5 males per vial, n = 8 vials per week). At 7, 30, or 50 days old, females were re-mated to 7 day-old males for 3 days. The 7 day-old progeny were then tested in the social space assay (15 flies/vial; separated by sex; 3 vials collected per week for 3 weeks for a total of n = 9 biological replicates).

### Behaviour assays

#### Handling prior to behaviour testing

Flies were tested in 3 internal replicates, on 3 separate days (trials were separated by at least one week, and up to 2 months) for a total of 9 biological replicates. On the day of each experiment, flies were transferred from treatment vials to fresh food vials and placed in the behaviour room to acclimate to the conditions (50% humidity, 25 °C) two hours prior to the experiment. The assays were performed between 12 pm and 3 pm (corresponding to 4 and 7 ZT - Zeitgeber time: the onset of light)^29^.

#### Social space assay

To quantify social space distances, we used an assay in which flies are forced into a group and must decide how close or far to settle away from others. The social space assay was performed as previously described^28,29^. In short, flies were acclimated to the behavioural room conditions (25 °C, 50% humidity) for two hours prior to being added via aspiration to a vertical arena (13–15) flies per arena, separated by sex, 3 replicates per week for 3 weeks for a total of n = 9 biological replicates and ~130 flies total). Images of the arenas were taken at ~30 minutes when flies had settled^28^. The images were analysed using the free software ImageJ (National Institutes of Health, Bethesda, Maryland, United States) to get the distance from each fly to its nearest neighbour, which is a variable recently used in several other studies^27,32,33,35^.

Behaviour of parents and progeny at increased temperature: Flies reared at 29 °C were tested with the social space assay at 7, 14, 21, and 30 days of age (13–15 flies/ arena, separated by sex, n = 9 biological replicates^28^). Flies reared to 7 days at 29 °C laid eggs on food that was then put on and kept at 25 °C, to rear the offspring to adulthood. The progeny (first generation) was then aged to 7 days and tested with the social space assay and compared to the 7 day-old progeny of parents aged to one week at 25 °C (13–15 flies/ arena, separated by sex, 3 replicates per week for 3 weeks for a total of n = 9 biological replicates).

Behaviour of parents after exposure to oxidative stress (Paraquat): 6 day-old flies were starved for 6 hours then fed 0 mM or 20 mM of paraquat in a solution of 5% sucrose and 1% blue dye (Club house^®^) for 13.5 hours before addition to the social space assay (note that a funnel was used here, not a mouth aspirator, to avoid exposure to paraquat). The social space assay and analysis were then performed as previously described (separated by sex, 13–15 flies/arena, 3 replicates per week for 3 weeks for a total of n = 9 biological replicates^28^).

Behaviour of progeny after exposure of parents to oxidative stress (paraquat): 6 day-old flies were starved for 6 hours then were fed either 0 mM or 20 mM paraquat for 13.5 hours as described above. Flies in mixed sex were then transferred to bottles containing Jazz-Mix^TM^ media for 2–3 days before removal. The eggs in the bottles were allowed to develop to adulthood prior to testing with the social space assay (separated by sex, 7 day-old, 13-15 flies/arena, 3 replicates per week for 3 weeks for a total of n = 9 biological replicates^28^).

Social space of parents of flies on caloric restriction: Individuals aged to 7 or 30 days on CR or 7 days on regular Jazz-Mix^TM^ media were tested in the social space assay as described above (separated by sex, 7 day-old,15 flies/ arena, 3 replicates per week for 3 weeks for a total of n = 9 biological replicates^28^). We were unable to test the social space of individuals aged to 50 days on CR because not enough flies survived to 50 days, although these flies were fertile and were able to lay enough progeny to test in social space.

Social space of progeny of flies on caloric restriction: Individuals aged to 7 or 30 days on CR were transferred to regular Jazz-Mix media for 3 days before parents were removed. The resulting progeny, which developed on regular food, were aged to 7 days and tested with the social space assay (separated by sex, 13–15 flies/ arena, 3 replicates per week for 3 weeks for a total of n = 9 biological replicates).

#### dSO avoidance assay

The dSO avoidance assay was performed as previously described^36^ in a binary choice apparatus called the T-maze. In this assay, flies choose to either enter a vial that previously contained stressed flies that emitted the dSO, or an empty vial^36,37^. We tested responder flies aged to 7, 14, 21 and 30 days, and the progeny of 7 and 30 days old parents (3 replicates per week for 3 weeks for a total of n = 9 biological replicates, 20 flies per vial, separated by sex). The emitter flies (those stressed and emitting dSO) were all 7 days old (20 flies per vial, equal mixed sex, stressed 1 minute via vortex). Responder and emitter flies were collected around 24 hours prior to the assay under cold anaesthesia.

Responders were permitted to choose the vial that previously contained stressed flies, the empty vial, or to not enter either (no choice). The number of flies that entered each vial or did not make a choice was then recorded. The performance index was calculated as follows:

$$\,\frac{(\#{\boldsymbol{flies}}\,{\boldsymbol{in}}\,{\boldsymbol{clean}}\,{\boldsymbol{vial}}-\,\#{\boldsymbol{flies}}\,{\boldsymbol{in}}\,{\boldsymbol{stressed}}\,{\boldsymbol{vial}})}{({\boldsymbol{total}}\,\#\,{\boldsymbol{of}}\,{\boldsymbol{flies}})}$$

#### EthoVision XT system to track distance moved by individual flies

We studied locomotion of aged flies and their progeny in an open field assay, as previously described (2–3 replicates per week for 3 weeks for a total of n = 8 biological replicates)^35^. Individual flies were aspirated into the arena (5.80 cm × 0.7 cm Petri dish on a white background and even lighting) and were acclimated for 1 minute before slight mechanical stimulation and placed under a Canon EOS Rebel T5 DSLR camera, which was mounted on a tripod. Videos were recorded for 5 minutes. All videos were converted to mpeg files and the total distance travelled (to determine the general activity level of each fly) was gathered for each fly and analysed with the Noldus EthoVision XT system (Noldus Information Technology, Netherlands).

### Microscopy

#### Male flies testes morphology and quantification of sperm bundles

Males at 7, 30, and 50 days of age were submerged in testes buffer (deionized water, 183 mM KCl, 47 mM NaCl, 10 mM Tris-HCl; n = 10 males per age group: 7 and 30 day-old males) on a glass dish under a dissection microscope (Nikon SMZ1500). Testes and surrounding accessory gland tissues were assessed for gross testes morphology and the number of sperm bundles were counted before adding DAPI solution (0.2% mg/ml) to visualize the sperm heads^62^. The number of bent versus the number of straight sperm heads was also quantified.

#### Persistence of sperm in the female flies reproductive tract

Reproductive tracts of females aged 0, 1, 2, 3, and 4 weeks post-mating with males expressing GFP-tagged sperm were placed in testes buffer (described above) to visualize the presence of sperm after the mating event. The spermatheca and seminal tubules were imaged using a standard fluorescence microscope within 1 hour post-dissection as a qualitative control to assess sperm ejection from female flies.

### Statistical tests

#### The social space assay

Past studies^28,29,35^ have shown that the distribution of the distances between flies does not follow a normal distribution. As such, the medians of the distances tend to be reproducible, but not the means, due to a few flies that may or may not stray away from the group. We thus first analysed the flies’ distribution using non-parametric tests (Kruskall-Wallis). Such tests have reduced power, and limit the type of comparison that can be made between or among treatment groups. So, in this study, to increase statistical power for our comparison, we normalized the data. We did so by removing these few outlier flies, when present, using a gentle ROUT (robust regression and outlier removal) on the original data by fitting the data to a model with a robust method in which outliers do not impact the fitting to the model^63^. The outliers were identified through the false discovery rate (FDR) where the value used, Q, is set to its lowest rate (Q = 0.1%) and only data points that were very far from the rest of the data (as predicted by the model) were removed as definitive outliers. Each treatment led to the generation of one mean of the distances. We averaged the 9 means obtained from the 9 biological replicates, and confirmed using a D’Agostino & Pearson normality test that those means were normally distributed. These means of mean distances were analysed with the statistical program GraphPad Prism (version 7.00 for Mac, GraphPad Software, La Jolla California USA, www.graphpad.com) to assess main effects using a one way-ANOVA with a Holm-Sidak *post hoc* test to correct for multiple comparisons in groups larger than two, or an unpaired t-test for groups of two (all measurements expressed as a mean ± standard error of the mean). We also used a two-way ANOVA and Holm-Sidak *post hoc* test to look at the interaction between groups such as sex and age. It is important to note that there was no difference in the statistical outcome of using non-parametric tests on the original data compared to using the parametric tests after normalisation.

#### dSO avoidance assay

Performance indices were compared using one-way or two-way ANOVA with Holm-Sidak *post hoc* tests.

#### EthoVision XT system

An unpaired student t-test was performed to compare 7 day-old males or females to either 30 day-old males or females or the 7 day-old progeny of parents aged to 30 days to compare distances travelled in an open field assay.

#### Straight and bent sperm heads

The number of straight and bent sperm heads of 7 and 30 day-old males were analysed with a two-way ANOVA and a Holm-Sidak *post hoc* test using GraphPad Prism7.

#### Representation of statistical significance on the figures

We indicate through asterisks either the results of the t-tests, the post-tests after a one-way ANOVA or write down the statistically significant main effect of a two-way ANOVA, as relevant for the comparisons we make in the text.

### Data availability

All data generated were analysed and presented in this article. The raw data and all reagents are available upon request.

### Ethics statement

Does not apply to the study of invertebrate animals including insects, as specified by Western’s Animal Care Committee or Ontario Provincial and Federal regulatory bodies.

## Electronic supplementary material

Supplemental data
