# Supplementary material for: Progeny of old parents have increased social space in Drosophila melanogaster
Source: Sci Rep. 2018 Feb 27;8:3673. doi: 10.1038/s41598-018-21731-0 (PMC5829228; doi:10.1038/s41598-018-21731-0)

Progeny of old parents have increased social space in *Drosophila melanogaster*: Supplemental Information

Authors.

Dova B. Brenman-Suttner[1], Shirley Q. Long[2], Vashine Kamesan[1], Jade N. de Belle[1], Ryley T. Yost[1], Rachelle L. Kanippayoor[1], Anne F. Simon[1]*.

[1]Department of Biology, University of Western Ontario, London, ON, Canada

[2]Department of Physiology and Pharmacology, University of Western Ontario, London, ON, Canada

Supplemental results and interpretation

Survival curve of our control strain. We performed survival curves of Canton-S, our laboratory strain, under standard conditions (25°C, 50% humidity, 12 hour day-night cycle, mixed male and female populations; **Supplemental Fig 1A**). We can differentiate 3 phases of aging. First a phase with no death: during that phase, we used 7 day-old flies as our "young" control, compared to 14 and 21 day-old individuals. Indeed, during this phase of aging (one-to-three weeks old), some behaviour such as locomotion, phototaxis and geotaxis are known to decline in performance[20,24], but flies did not die of aging and fertility (number of eggs laid) rates were fairly constant (Fig 1A). Specifically, at 1-4 days old there was a high fertility 3.3 eggs/per female per day. At 6-8 days old, there was a plateau with only 0.1 egg/per female per day, but 1.2 eggs/per female per day at 14 and 21 days. In the second phase of aging (4-to-7 weeks), fertility started to decrease (0.3 egg/per female per day at 30 days old, and 0.2 at 50 days old). We were able to study flies during that stage, which was at 90% alive (30 days old), and 50% alive (50 days old). Beyond that age, we reached a phase (over eight weeks old) during which no more progeny can be collected and individuals display their most pronounced decline in behavioural performance[20,24].

Thus, in our hands, *the flies* survival and fecundity in aging parents were shown to decline after 30 days, much like other studies[1–3]. Female age can affect oogenesis via GSC exhaustion, changes in hormone-sensing, less effective cellular machinery, damaged proteins and via epigenetic changes[4,5]. These mechanisms may account for changes in egg production and a reduction in fecundity (progeny viability) with aging.

Increased survival, and decreased fecundity and fertility in the progeny of old parents

Survival. To identify how parental aging would affect life history traits such as survival, fertility (number of eggs laid) and fecundity (number of adults that arise from eggs laid) of the progeny (first generation, G1) and grand-progeny (second generation, G2) we generated several curves (**Supplemental Fig 1B**). We also extracted the number of eggs laid and progeny that developed from those eggs at 100%, 90%, and 50% survival for

each generation (**Supplemental Fig 1D**). The 3 generations tested had statistically different survival curves in a Log-rank (Mantel-Cox) test: X^2 (2, n=641) = 271.3, p<0.0001. Each of the survival curves differed from that of the control (G1: Log-rank (Mantel-Cox) test: X^2 (1, n=458) = 23.85, p<0.0001, G2: Log-rank (Mantel-Cox) test: X^2 (1, n=428) = 165.5, p<0.0001). Specifically, 7 day-old progeny of 30 day-old flies lived longer than the progeny of 7 day-old flies. As behaviour was restored in the second generation of 30-day-old flies, we also wanted to test their life history traits. Flies with old grandparents lived shorter, but had normal fecundity (although their fertility was lower; **Supplemental Fig 1B**).

Additionally, differences in survival among the three groups (control, first generation, and second generation of aged parents) were significantly different after each group reaches 50% survival (two-way ANOVA with a Holm-Sidak *post hoc* test; **Supplemental Fig 1D**). The maximum survival also significantly differed among the three groups (**Supplemental Fig 1D**). Age ($F_{2,16}$=44.04; p<0.0001), generation (control, first, second; $F_{6,16}$=12.97; p<0.0001) and their interaction ($F_{3,16}$=220.78; p<0.0001) were each significant in the two-way ANOVA and Holm-Sidak *post hoc* test.

Fertility and fecundity. The differences among cumulative fertility (number of eggs laid; **Supplemental Fig 1E**) and fecundity (number of adult progeny; **Supplemental Fig 1F**) were not different at 100% and 90% survival. However, the fertility and fecundity at 50% survival of the control were significantly different from the first generation (p<0.01) and second generation (p<0.001) of old parents.

The fecundity and fertility of the first generation of old parents were not reduced but longevity was increased, as compared to the progeny of young parents and the second generation of old parents. Similar aging studies have shown a different effect, where parental age results in reduced longevity, although those studies have looked at the effect of only one aged parent[7]. Some studies have shown that the first generation of parents that have been exposed to a stressor (here, aging) have increased longevity and reduced fertility and fecundity as a mode of stress resistance by diverting energy away progeny production[8]. However, this trade-off was not seen as the progeny production of the first

generation of old flies was increased and was maintained in the second generation of aged parents. Therefore, it is possible that the aging stressor was not a powerful enough force to induce this strong trade-off with longevity and fecundity, although behaviour was affected. Interestingly, the second generation of aged parents had reduced longevity but maintained fertility and fecundity and did not have a change in social space.

Supplemental methods

Survival Curves

Standard conditions: Flies were collected from stock bottles under cold anaesthesia at 2-3 days old (40 mixed-sex flies per vial, 25°C, 50% humidity with a 12:12 light: dark cycle). We tested 3 independent replicates on 3 separate occasions (trials were separated by at least one week, and up to 2 months) for a total of 9 biological replicates. Flies were transferred to new food every 2-3 days and the number of dead flies was counted. The resulting survival curve was generating using Microsoft Excel. A curve combining all 9 replicates was used as the basis for all subsequent studies (Supplemental Fig 1A,D)

Increased temperature: A survival curve of flies at 29°C was generated as those at 25°C (above). Flies were transferred to new food every 2-3 days, the number of dead flies was recorded, and flies were always placed back into the 29°C incubator (Supplemental Fig 3A).

Exposure to oxidative stress (Paraquat): As previously described[61], Canton-S flies were aged to 6 days in mixed sex, then separated by sex, and starved for 6 hours in empty vials. 3 vials collected containing 5 males or females per vial were collected per week for 3 weeks for a total of n=9 vials per concentration of Methyl Viologen (Sigma Aldrich, St. Louis, Missouri, USA; hereby known as Paraquat); and maintained at 25°C, 50% humidity 12:12 light: dark cycle, 8 am lights on. Flies were then administered either 0mM, 10mM, 20mM or 40mM of Paraquat in a solution of 5% sucrose and 1% blue dye (club house®) to confirm food consumption as the dye was visible in the gut of the fly[62]. The solution was added to Whatman®3 filter paper (Sigma Aldrich, 500 µl/ paper) in empty vials and those papers with aliquots of sucrose solution were replaced every 24 hours. The number of dead flies in each vial was counted every 2 hours until all flies

were dead in the 10mM, 20mM, and 40mM conditions. The number of dead flies in the 0mM condition was then measured once each day until all flies were deceased. We chose to use 20mM for future experimentation as the time at which flies reach 90% survival was after 13.5 hours, which permitted fly preparation one day before experimentation (Supplemental Fig 3B)

Caloric restriction. Survival curve of flies on caloric restriction (CR) were generated as those on Jazz Mix Media (above; Supplemental Fig 3B). Flies were transferred to new CR food every 2-3 days; the number of dead flies was recorded.

Fertility and fecundity curves

Fertility and fecundity throughout life-span: Fertility and fecundity curves were performed as previously described[61]. Fertility is measured here as the number of eggs laid per female over the course of the flies' lifespan. Fecundity, however, is a measure of the number of progeny that arise from the eggs laid.

Fertility and fecundity of aging mothers: To determine when females stop laying fertile eggs and can then be re-mated with young males, we performed a fertility and fecundity curve on aging females isolated from males. The majority of sperm are no longer visible in the female fly reproductive tract after three weeks' separation from male flies (Fig 2E). After being mated for three days and subsequently isolated from males, female flies lay eggs continuously throughout their lives as shown by a steadily increasing cumulative number of eggs per day per female (Supplemental Fig 3E). The majority of these eggs yield adults until approximately 25 days, at which point the cumulative mean number of adults eclosing per day per female plateaus. Females were then re-mated at 30 days with young males and the progeny were assessed.

Fertility and fecundity of both aged parents: Virgin females were collected with young males (< 2 days old) and maintained over Jazz Mix media containing several drops of blue food dye (club house®) for contrast to visualize eggs (5 males and 5 females per vial, 3 vials collected per week for 3 weeks for a total of 9 biological replicates). Flies were transferred daily into fresh vials and the number of eggs laid per day was counted daily to quantify fertility. The number of dead males and females were also quantified for later calculations of the number of eggs and progeny laid per female. Fecundity was assessed

by counting the resulting progeny ~11 days later, when they emerge as adults. Both fertility and fecundity are average values per female over the 9 biological replicates and are represented as cumulative values over time. The resulting cumulative curves of fecundity were generated using Microsoft Excel.

Statistics on fertility and fecundity

Log-Rank (Mantel-Cox) Pairwise Comparison test was performed to compare survival distributions between groups or pairs of curves. The values of cumulative fertility (number of eggs laid per female) and cumulative fecundity (egg-to-adult viability) were extracted for 100%, 90%, 50% and maximum survival. These values were placed into GraphPad Prism7 and a two-way ANOVA and a Holm-Sidak *post hoc* test to compare each group.

Supplemental References:

1. Hu, Y., Han, Y., Wang, X. & Xue, L. Aging-related neurodegeneration eliminates male courtship choice in Drosophila. *Neurobiol. Aging* **35,** 2174–2178 (2014).
2. Simon, A. F., Shih, C., Mack, A. & Benzer, S. Steroid control of longevity in *Drosophila melanogaster*. *Science* **299,** 1407–1410 (2003).
3. Simon, A. F., Liang, D. T. & Krantz, D. E. Differential decline in behavioral performance of *Drosophila melanogaster* with age. *Mech. Ageing Dev.* **127,** 647–651 (2006).
4. Dailey, T., Dale, B., Cohen, J. & Munné, S. Association between nondisjunction and maternal age in meiosis-II human oocytes. *Am. J. Hum. Genet.* **59,** 176–184 (1996).
5. Zhao, R., Xuan, Y., Li, X. & Xi, R. Age-related changes of germline stem cell activity, niche signaling activity and egg production in Drosophila. *Aging Cell* **7,** 344–354 (2008).
6. Hercus, M. J. & Hoffmann, A. A. maternal and grandmaternal age influence offspring fitness in Drosophila. *Proc. Biol. Sci.* **267,** 2105–2110 (2000).
7. Priest, N. K., Mackowiak, B. & Promislow, D. E. L. The role of parental age effects on the evolution of aging. *Evolution (N. Y).* **56,** 927–935 (2002).
8. De Loof, A. Longevity and aging in insects: Is reproduction costly; cheap; beneficial or irrelevant? A critical evaluation of the 'trade-off' concept. *J. Insect Physiol.* **57,** 1–11 (2011).

Supplemental figure legends

Supplemental Figure 1. The progeny of 30 day-old parents has increased longevity and reduced fecundity, which is not fully recovered in the second generation. (A) Survival and fertility curves of the progeny of seven-day-old parents. We choose 7 days to represent 100% survival, 90% and 50% survival are seen respectively at 30 ± 1.53 and 52.3 ± 7.51 days. The maximum survival is 91.3 ± 2.60 days. **(B) Same curves as in A, compared to survival and fertility of G1 and G2.** Survival is reported on left axis, cumulative fertility is shown on the secondary axis. The survival of the 3 generations are statistically different: Log-rank (Mantel-Cox) test: X^2 (2, n=641) = 271.3, p<0.0001 **(C) Same curves as in A, compared to survival and fecundity of G1 and G2**. G1 and G2 display increase fecundity. **(D) Differences among survival** only arise when comparing 50% survival: parental generation has a mean survival 52.3±7.51 days, and G1 (66.5 ± 0.50 days) are not statistically different, but G2 (33±8.00 days) is significantly different from both parental and G1. The maximum (max) survival of the parental (91.3±2.60 days) is significantly different from both G1 (148±9.00 days) and G2 (64±1.00 days). **(E) The fertility among the different generations** was again only significantly different at 50% survival. Parental flies laid fewer eggs per female (102.08±11.82 eggs) by 50% survival, than G1 (161.92 ± 25.90) and G2 (161.62±21.77). Parental flies also had reduced maximum fertility per female (112.51±8.67) relative to G1 (164.45 ± 27.25) and G2 (202.02 ± 31.67). **(F) Similarly, at 50% survival, parental flies had fewer adult progeny per female** (56.92±5.09) than G1 (96.76±14.48) or G2 (102.73±14.25). Parental flies also had fewer total maximum progeny per female (57.52±4.91) as compared to G1 (101.82 ± 16.14) and G2 (105.81±13.68).
(D-E) n=9; two-way ANOVA with a Holm-Sidak *post hoc* test.
For all graphs, n= number of replicates, ns= not significant, *p< 0.05, **p< 0.01, ***p< 0.001, and ****p< 0.0001

Supplemental Figure 2. Parental aging effects on social space do not extend beyond the second generation.

(A-C): The mean distance of each fly to its nearest neighbour (mean± s.e.m.) in the social space assay (n=9; unpaired, one-tailed student t-test**). No effect of age from the parental generation on the third through the fifth generation: (A):** third generation (G3); **(B)** fourth generation (G4); **(C)** fifth generation (G5) .

n= number of replicates, **for all graphs,** ns= not significant, *$p < 0.05$, **$p < 0.01$, ***$p < 0.001$, and ****$p < 0.0001$

Supplemental Figure 3. Environmental manipulations of the flies affect both survival and fecundity.

(A) Increased temperature results in shorter lifespan and reduced fecundity (n=9). Survival, fertility, and fecundity were quantified to determine if lifespan and progeny production were affected by changes to environmental conditions. Flies at 29°C reached 100% survival on the first day of quantification and thus there was no cumulative fecundity or fertility that could be quantified on the first day of data collection. They reached 90% survival at 11.67±8.29 days, 50% survival at 35.33±4.32 days and maximum survival at 58.67±4.32 days. At 90% survival, females laid an average of 36.05±6.83 eggs and 9.10±2.57 progeny, by 50% survival, females yielded 72.64±10.60 eggs and 16.14±3.75 progeny, and by maximum survival, females have laid 77.75±11.81 eggs and 17.24±3.50 progeny. Survival, fecundity, and fertility were each reduced relative to control (see Supplemental Figure 1). **(B) Survival curves of flies fed Paraquat.** Flies were fed either 0mM, 10mM, 20mM or 40mM of paraquat (n=9 each). Flies fed 0mM paraquat survived up to 10 days when the glucose solution was provided once daily. When flies were fed 10mM paraquat, flies reached 100% survival at 12.33±1.05 hours, 90% at 13.5 ±1.19 hours, 50% at 37.83±8.16 hours and maximum survival at 131±14.29 hours. When flies were fed 20mM paraquat, flies reached these time points at 9.33±0.73 hours, 12.67±1.73 hours, 25.33± 1.99 hours and 82±9.13 hours. As both 10mM and 20mM fed flies reached 90% survival at around the same time, we chose to continue to feed flies 20mM paraquat as this concentration was used in prior aging studies to induce oxidative stress. Finally, flies fed 40mM paraquat reached 100%

survival at 9.17±2.01 hours, 90% at 12.33±0.73 hours, 50% at 18.5±2.15 hours, and maximum survival at 75.33±15.65 hours. **(C) Flies fed caloric restriction (CR) do not live longer but lay more eggs and yield more progeny than those on regular food (Figure 1A, C-E; n=9).** Flies on CR reached 100% survival after 2.67±0.89 days, 90% survival at 22.33±7.63 days, 50% survival at 32.33±11.74 days, and maximum survival was reached at 54.00±5.10 days. However, maximum cumulative egg laying and maximum cumulative fecundity are increased relative to those on regular food. At 100% survival there were 4.83±1.91 eggs and 3.72±2.02 progeny, at 90% survival there were 243.96±33.95 eggs and 65.72±15.89 progeny, at 50% survival there were 368.12±50.30 eggs and 89.67±16.07 progeny, and at maximum survival there were 446.08±72.44 eggs and 105.96±14.19 progeny. **(D) Fertility and fecundity following mating events in aging females (n=5).** Females were mated with males containing a GFP tag on their sperm for several days, which are then removed (A) to allow females to age in the absence of males or re-mating. Females stopped laying fertile eggs around 22 days (B), at which point females could be re-mated and the paternity of the progeny could be certain (as evident as eggs are still being (light blue) laid but the cumulative progeny (dark blue) plateaus). The "7d M" indicates when re-mating of the female took place with young males that do not contain GFP tagged sperm. **(E) Survival of different strains of D. melanogaster (n=9).** Oregon-R (light grey) reached 100% survival at 1.50 ±0.58 days, 90% survival at 17.25±3.72 days, 50% survival at 34.75±3.07 days, and maximum survival at 57.75±6.47 days. Elwood (dark grey) reached 100% survival at 1.50±0.58 days, 90% survival at 15.50 ±0.58 days, 50% survival at 40.50±2.24 days and maximum survival at 58.25±5.89 days.

Supplemental Figure 1

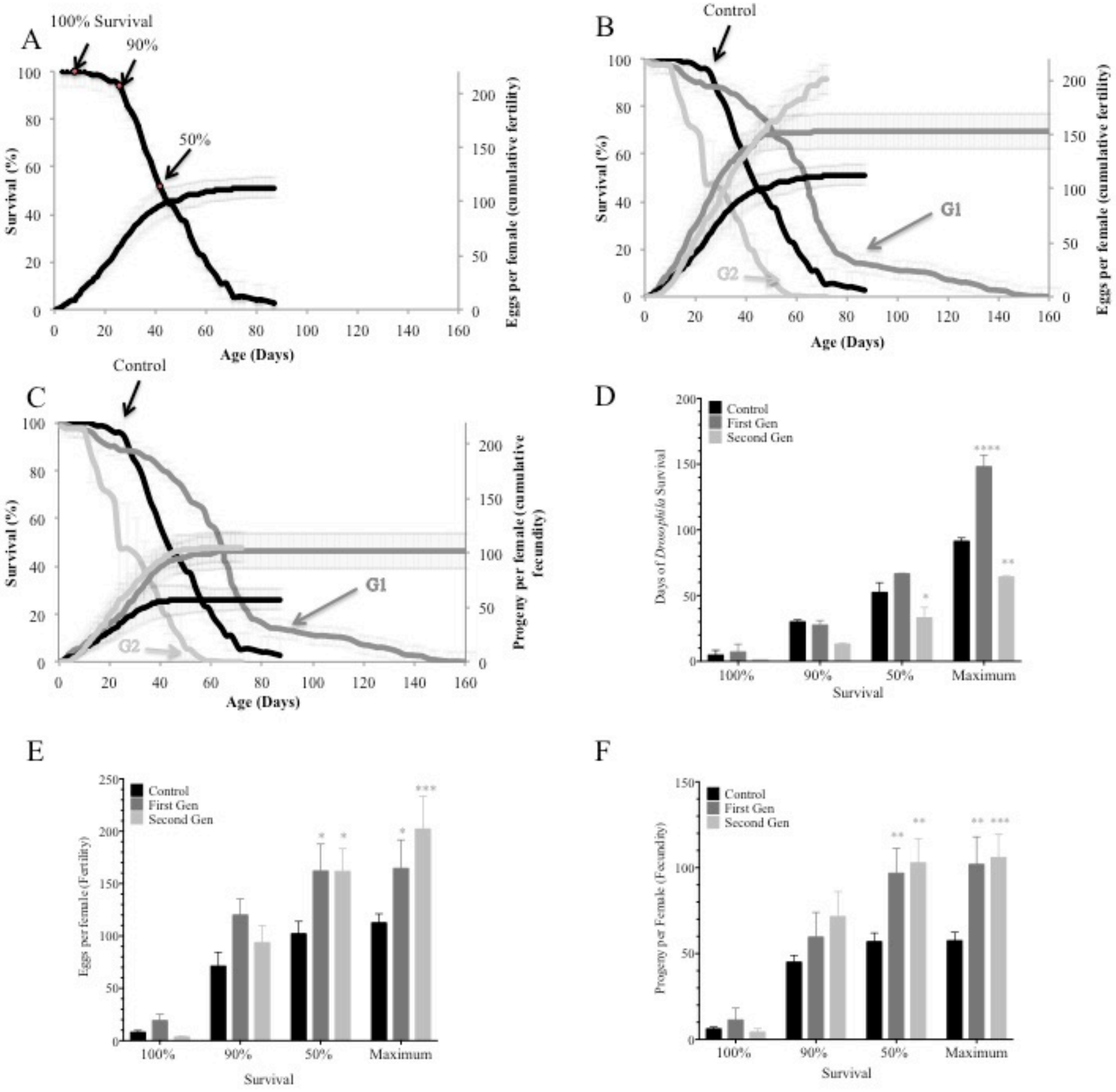

Supplemental Figure 2

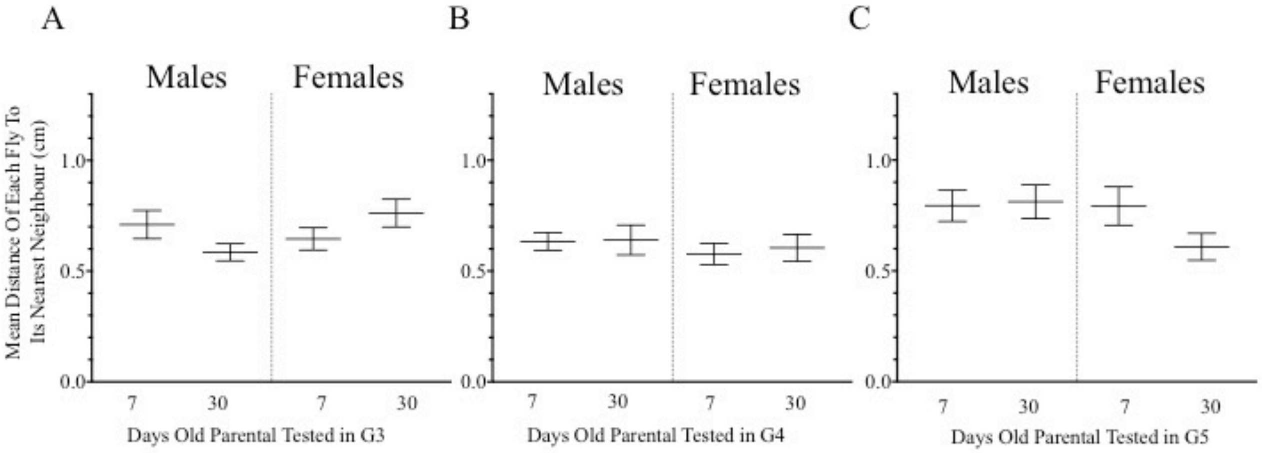

Supplemental Figure 3

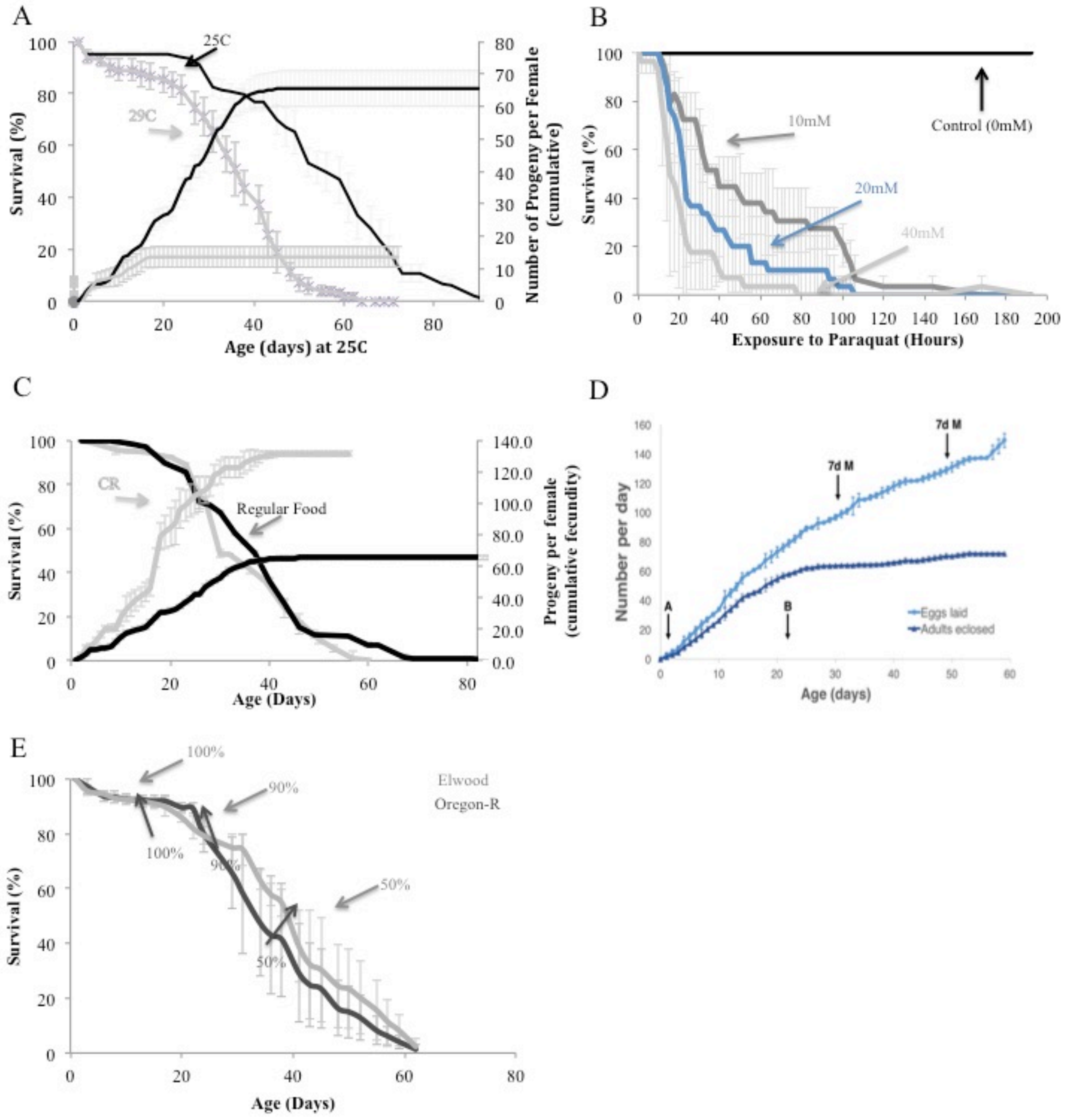

Supplement: Supplementary file 1 — Supplemental data [file 41598_2018_21731_MOESM1_ESM.pdf]
